# Associations between first-trimester screening biomarkers and maternal characteristics with gestational diabetes mellitus in Chinese women

**DOI:** 10.3389/fendo.2024.1383706

**Published:** 2024-08-08

**Authors:** Yu-Ting Lu, Chie-Pein Chen, Fang-Ju Sun, Yi-Yung Chen, Liang-Kai Wang, Chen-Yu Chen

**Affiliations:** ^1^ Department of Obstetrics and Gynecology, MacKay Memorial Hospital, Taipei, Taiwan; ^2^ Department of Medical Research, MacKay Memorial Hospital, Taipei, Taiwan; ^3^ Department of Medicine, MacKay Medical College, Taipei, Taiwan

**Keywords:** gestational diabetes mellitus, first-trimester biomarkers, maternal characteristics, pregnancy-associated plasma protein A, placental growth factor

## Abstract

**Background:**

Gestational diabetes mellitus (GDM) can result in adverse maternal and neonatal outcomes. Predicting those at high risk of GDM and early interventions can reduce the development of GDM. The aim of this study was to examine the associations between first-trimester prenatal screening biomarkers and maternal characteristics in relation to GDM in Chinese women.

**Methods:**

We conducted a retrospective cohort study of singleton pregnant women who received first-trimester aneuploidy and preeclampsia screening between January 2019 and May 2021. First-trimester prenatal screening biomarkers, including pregnancy-associated plasma protein A (PAPP-A), free beta-human chorionic gonadotropin, and placental growth factor (PLGF), along with maternal characteristics, were collected for analysis in relation to GDM. Receiver operating characteristic (ROC) curve and logistic regression analyses were used to evaluate variables associated with GDM.

**Results:**

Of the 1452 pregnant women enrolled, 96 developed GDM. PAPP-A (5.01 vs. 5.73 IU/L, *P* < 0.001) and PLGF (39.88 vs. 41.81 pg/mL, *P* = 0.044) were significantly lower in the GDM group than in the non-GDM group. The area under the ROC curve of combined maternal characteristics and biomarkers was 0.73 (95% confidence interval [CI] 0.68–0.79, *P* < 0.001). The formula for predicting GDM was as follows: *P* = 1/[1 + *exp* (-8.148 + 0.057 x age + 0.011 x pregestational body mass index + 1.752 x previous GDM history + 0.95 x previous preeclampsia history + 0.756 x family history of diabetes + 0.025 x chronic hypertension + 0.036 x mean arterial pressure - 0.09 x PAPP-A - 0.001 x PLGF)]. Logistic regression analysis revealed that higher pregestational body mass index (adjusted odds ratio [aOR] 1.03, 95% CI 1.01 - 1.06, *P* = 0.012), previous GDM history (aOR 9.97, 95% CI 3.92 - 25.37, *P* < 0.001), family history of diabetes (aOR 2.36, 95% CI 1.39 - 4.02, *P* = 0.001), higher mean arterial pressure (aOR 1.17, 95% CI 1.07 - 1.27, *P* < 0.001), and lower PAPP-A level (aOR 0.91, 95% CI 0.83 - 1.00, *P* = 0.040) were independently associated with the development of GDM. The Hosmer-Lemeshow test demonstrated that the model exhibited an excellent discrimination ability (chi-square = 3.089, df = 8, *P* = 0.929).

**Conclusion:**

Downregulation of first-trimester PAPP-A and PLGF was associated with the development of GDM. Combining first-trimester biomarkers with maternal characteristics could be valuable for predicting the risk of GDM.

## 1 Introduction

Gestational diabetes mellitus (GDM) is typically diagnosed between 24 and 28 weeks of gestation, though in some countries, screening and diagnosis can also take place as early as 16 to 18 weeks. It is a major medical complication characterized by glucose intolerance during pregnancy, and it can result in adverse maternal and neonatal outcomes. Women diagnosed with GDM are at an increased risk of developing preeclampsia and the necessity of undergoing a cesarean delivery, as well as developing chronic diabetes mellitus (DM) and cardiovascular disease in subsequent years (1). In addition, the risks of neonatal complications such as macrosomia, birth trauma, neonatal hypoglycemia, and hyperbilirubinemia are also increased (1). Children born to mothers with GDM are also susceptible to an increased risk of obesity and the subsequent development of chronic DM in their adult years (2). The global prevalence of GDM has been reported to range from 2.2% to 37.9% using the Carpenter and Coustan criteria or the National Diabetes Data Group (NDDG) criteria, and from 3.5% to 38.6% using the International Association of Diabetes and Pregnancy Study Groups (IADPSG) criteria (3). The prevalence of GDM continues to increase due to increases in maternal age and obesity (1). Earlier dietary interventions and mild-moderate exercise can reduce the risk of GDM (4); however, GDM is not usually diagnosed until late in the second trimester, at which point it is too late to prevent its development.

First-trimester aneuploidy screening has been performed for more than two decades (5). A combination of maternal age and history, fetal nuchal translucency thickness, and levels of maternal serum pregnancy-associated plasma protein A (PAPP-A) and free beta-human chorionic gonadotropin (β-hCG) in the first trimester has been shown to be able to detect about 90% of cases of trisomy 21 (5). Moreover, incorporating additional ultrasound markers, including fetal nasal bone, tricuspid valve flow, and ductus venosus flow has been shown to enhance the detection rate to 95% (6). In addition, first-trimester preeclampsia screening using a combination of maternal risk factors, mean arterial pressure (MAP), uterine artery pulsatility index (PI), and maternal serum PAPP-A and placental growth factor (PLGF) levels has been demonstrated to achieve 93% and 36% detection rates for early-onset and late-onset preeclampsia, respectively (7).

Previous studies have investigated the association between first-trimester PAPP-A and/or free β-hCG levels and GDM development, however the results have not been consistent across studies (**Table 1**) (8–27). Furthermore, few studies have investigated the association between first-trimester PLGF level and the subsequent development of GDM, and the results have also been inconsistent (17, 18, 25, 26). First-trimester biomarkers are also influenced by the ethnic origin of the women (28, 29), however few studies have discussed first-trimester PAPP-A and free β-hCG levels related to the development of GDM in Chinese women (20, 23), and no study has investigated the association between first-trimester PLGF level and GDM in a Chinese population. Since Chinese women have been shown to have a high risk of GDM (30), the objective of this study was to investigate the association between first-trimester prenatal screening biomarkers and the development of GDM in a cohort of Chinese women, and then further combine these biomarkers with maternal characteristics for a comprehensive analysis to clarify whether this could improve the predictive ability for the development of GDM.

**Table 1:** Studies related to first-trimester prenatal screening biomarkers and GDM.

Study	Number		PAPP-A		Free β-hCG		PLGF	
	GDM	Non-GDM	GDM	Non-GDM	GDM	Non-GDM	GDM	Non-GDM
Ong (2000) (8)	49	4297	0.848 (0.691-1.006)* Median (95% CI)	1.049 (1.028-1.070) Median (95% CI)	0.783 (0.587-0.979)* Median (95% CI)	1.010 (0.984-1.036) Median (95% CI)	–	–
Tul (2003) (9)	27	1109	0.98	1.01	0.86	0.99	–	–
Beneventi (2011) (10)	228	228	0.7 (0.5-1.2)*	1.2 (0.8-1.6)	0.9 (0.6-1.6)	1.0 (0.7-1.5)	–	–
Savvidou (2012) (11)	779	41007	0.94 (0.65-1.39)	1.00 (0.68-1.42)	0.95 (0.64-1.51)	1.00 (0.68-1.52)	–	–
Husslein (2012) (12)	72	216	1.17 ± 0.71	1.13 ± 0.58	1.13 ± 0.73	1.15 ± 0.64	–	–
Spencer (2013) (13)	870	6559	0.91*	1.00	0.93*	1.00	–	–
Lovati (2013) (14)	307	366	0.9 ± 0.6*	1.3 ± 0.6	1 (0.7-1.6)	1.05 (0.7-1.6)	–	–
Kulaksizoglu (2013) (15)	60	60	0.77 (0.42)*	0.97 (0.40)	0.93 (0.53)	0.97 (0.29)	–	–
Beneventi (2014) (16)	112	112	1.06 (0.59)*	1.22 (0.64)	–	–	–	–
Eleftheriades (2014) (17)	40	94	-0.02 (0.19)* Log_10_ MoM	0.02 (0.20) Log_10_ MoM	0.0032 (0.29) Log_10_ MoM	0.0035 (0.24) Log_10_ MoM	1.76 (0.19)* Log_10_ MoM	1.68 (0.15) Log_10_ MoM
Syngelaki (2015) (18)	787	30438	0.949 (0.913-0.987)* Median (95% CI)	1.000 (0.994-1.006) Median (95% CI)	–	–	1.053 (1.023-1.083)* Median (95% CI)	1.000 (0.995-1.005) Median (95% CI)
Wells (2015) (19)	364	1282	Early: 0.79 (0.51-1.28)* Late: 0.94 (0.63-1.31)*	1.00 (0.68-1.40)	–	–	–	–
Cheuk (2016) (20)	169	351	0.97 (0.65-1.32)	0.99 (0.67-1.44)	1.05 (0.73-1.64)	1.02 (0.71-1.55)	–	–
Farina (2017) (21)	12	60	0.70 (0.55-1.04)*	1.10 (0.72-1.44)	–	–	–	–
Sweeting (2017) (22)	248	732	0.81 (0.58-1.20)*	1.00 (0.70-1.46)	0.98 (0.64-1.45)	0.99 (0.68-1.55)	–	–
Xiao (2018) (23)	599	986	0.88 (0.60-1.28)*	0.97 (0.67-1.37)	1.01 (0.69-1.58)	1.06 (0.73-1.62)	–	–
Visconti (2019) (24)	596	1828	1.02 (0.77-1.68)	1.19 (0.82-1.67)	1.02 (0.60-1.36)	0.91 (0.61-1.36)	–	–
Correa (2019) (25)	16	80	–	–	–	–	11 (18.1)	5.25 (11.15)
Tenenbaum-Gavish (2020) (26)	20	185	0.62 (0.49-0.77)*	1.01 (0.88-1.15)	–	–	1.09 (0.86-1.33)	1.00 (0.95-1.06)
Yanachkova (2022) (27)	412	250	1.2 (0.62)*	1.3 (0.65)	1.29	1.36	–	–
Our study	96	1356	0.88 (0.57-1.11)*	1.01 (0.70-1.40)	0.93 (0.63-1.50)	1.00 (0.68-1.51)	0.96 (0.65-1.21)*	1.00 (0.70-1.33)

Data are presented as median (interquartile range) or mean ± standard deviation.

GDM, gestational diabetes mellitus; PAPP-A, pregnancy-associated plasma protein A; β-hCG, beta-human chorionic gonadotropin; PLGF, placental growth factor; MoM, multiple of the median; CI, confidence interval.

*P < 0.05 was considered statistically significant.

## 2 Materials and methods

### 2.1 Study population

This retrospective cohort study was conducted at MacKay Memorial Hospital, a tertiary referral hospital in Taipei, Taiwan, from January 2019 to May 2021. The study consecutively enrolled pregnant Chinese women who underwent ultrasound and serum assessments for aneuploidy and preeclampsia screening during the first trimester (11 0/7 to 13 6/7 gestational weeks). The exclusion criteria were as follows: multiple pregnancies, pregestational DM or use of drugs affecting glucose homeostasis before and during pregnancy, current preeclampsia, other chronic systemic diseases excluding DM or cardiovascular disease, major fetal anomalies, and maternal age less than 18 or greater than 50 years. GDM was diagnosed between 24 and 28 gestational weeks according to the one-step (IADPSG) or two-step (Carpenter and Coustan criteria or NDDG) criteria, at the discretion of the attending physician. This study was approved by the Institutional Review Board of MacKay Memorial Hospital on 26 September 2022 (IRB no. 22MMHIS318e). All personal identifiers were anonymized prior to data collection.

### 2.2 Collection of maternal characteristics

Maternal characteristics included age, pre-gestational body mass index (BMI), nulliparity, previous history of GDM, previous history of gestational hypertension or preeclampsia, family history of DM (defined as chronic DM in a first- or second-degree relative), previous macrosomia (neonatal birth weight ≥ 4000 g), history of polycystic ovarian syndrome, *in vitro* fertilization, cardiovascular disease (such as stroke and myocardial infarction), and chronic hypertension. Gestational hypertension was established when there was new onset of elevated blood pressure (systolic blood pressure (BP) ≥ 140 mmHg and/or diastolic BP ≥ 90 mmHg), observed on at least two separate occasions with a minimum interval of 4 hours, originating at ≥ 20 weeks into gestation, and lacking concurrent proteinuria. Preeclampsia was defined according to the diagnostic criteria stipulated by the American College of Obstetricians and Gynecologists, involving the combination of gestational hypertension along with proteinuria or emerging indications of end-organ dysfunction (31). Chronic hypertension during pregnancy was characterized by hypertension initially detected at < 20 gestational weeks.

### 2.3 Collection of first-trimester prenatal screening data

Maternal blood samples, approximately 3 mL in volume, were collected and promptly dispatched to the laboratory. Upon centrifugation, the serum was separated and stored at -20°C until analysis. Maternal serum concentrations of PAPP-A, free β-hCG, and PLGF were quantified using a Kryptor analyzer (Brahms GmbH, Hennigsdorf, Germany).

Following a 15-minute rest period, the participants assumed a seated posture with their arms supported at heart level for BP measurements. BP was simultaneously recorded on both arms using adult cuffs, and a series of four consecutive readings were taken at 1-minute intervals. MAP was calculated by averaging the systolic BP and twice the diastolic BP from both arms, then dividing by three.

Ultrasound assessments were conducted using a Voluson E10 ultrasound system (GE Medical Systems, Zipf, Austria) equipped with a 3–9 MHz transabdominal probe. Gestational age was established by assessing the crown-lump length during the first trimester. The ultrasound procedures adhered to the protocols outlined by the Fetal Medicine Foundation, London, for evaluations of fetal nuchal translucency and maternal uterine artery PI measurements (http://www.fetalmedicine.com).

### 2.4 Statistical analysis

Statistical analyses were conducted using SPSS version 28.0 (IBM Corporation, Armonk, NY). Categorical variables were evaluated using the chi-square test and Fisher’s exact test. The Kolmogorov-Smirnov test was conducted to evaluate whether the continuous variables showed a normal distribution. Upon recognizing that the data did not adhere to a normal distribution, we subsequently employed the Mann-Whitney U test and presented the data using the median (interquartile range). To determine optimal cut-off values for continuous variables linked to GDM, receiver operating characteristic (ROC) curves and the Youden index were used. Univariate and multivariate logistic regression analyses were conducted to explore the confounding factors associated with GDM. The Hosmer-Lemeshow test was used to assess the goodness of fit of the logistic regression model. A significance level of *P* < 0.05 was considered statistically significant.

## 3 Results

### 3.1 Maternal characteristics of pregnant women

**Figure 1:**
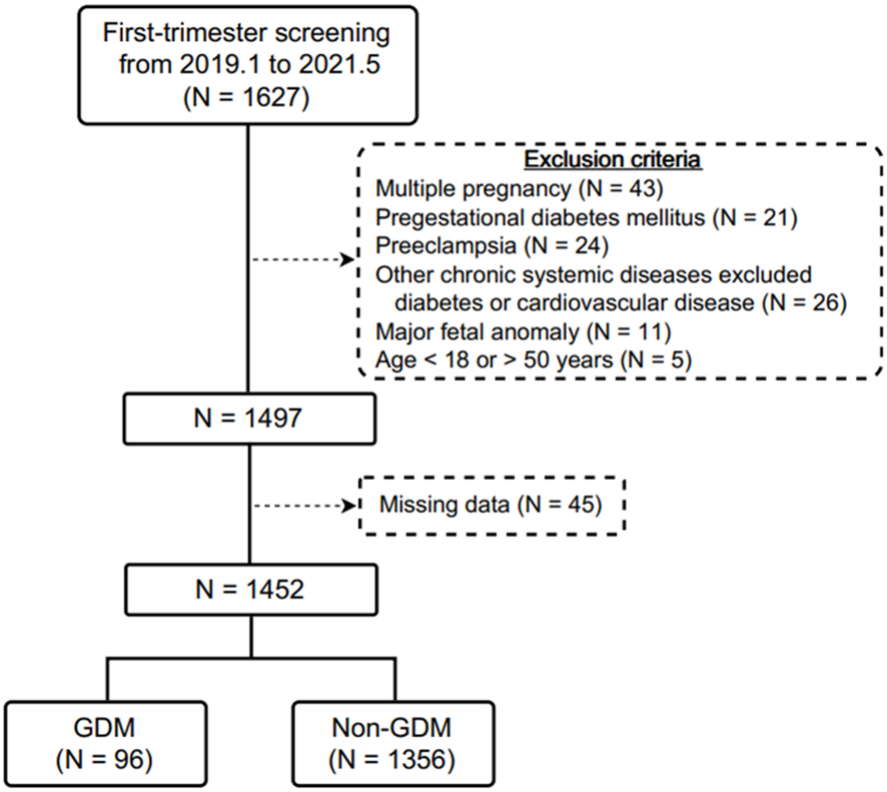
Flow diagram of the included pregnant women receiving first-trimester prenatal screening in this study.

During the study period, 1627 pregnant women were initially recruited during first-trimester prenatal screening examinations. After excluding those with multiple pregnancies (N = 43), pregestational DM (N = 21), current preeclampsia (N = 24), other chronic systemic diseases apart from DM or cardiovascular disease (N = 26), major fetal anomalies (N = 11), those aged < 18 or > 50 years (N = 5), and those without complete data (N = 45), 1452 participants were enrolled into this study, of whom 96 (6.61%) were diagnosed with GDM (**Figure 1**). Of these 96 women, 17 (17.71%) were diagnosed using the IADPSG criteria, and 79 (82.29%) using the Carpenter and Coustan or NDDG criteria.

**Table 2** shows comparisons of the maternal characteristics of the GDM and non-GDM groups. There were no significant differences in nulliparity, history of gestational hypertension, history of macrosomia, history of polycystic ovarian syndrome, *in vitro* fertilization, and cardiovascular disease between the two groups. However, significantly higher maternal age (33 vs. 32 years, *P* = 0.039), pregestational BMI (23.7 vs. 21.6 kg/m2, *P* < 0.001), and higher rates of previous GDM (11.5% vs. 0.8%, *P* < 0.001), previous preeclampsia (4.2% vs. 0.8%, *P* = 0.014), family history of DM (24.0% vs. 11.4%, *P* < 0.001), and chronic hypertension (5.2% vs. 1.2%, *P* = 0.010) were noted in the GDM group compared to the non-GDM group.

**Table 2:** Maternal characteristics.

	GDM (N = 96)	Non- GDM (N = 1356)	*P* value
Age (years)	33 (30-36.8)	32 (30-35)	0.039*
Pregestational BMI (kg/m^2^)	23.7 (21.2-28.5)	21.6 (19.7-24.0)	< 0.001*
Nulliparity	66 (68.8%)	930 (68.6%)	0.973
Previous GDM	11 (11.5%)	11 (0.8%)	< 0.001*
Previous gestational hypertension	0 (0%)	4 (0.3%)	> 0.999
Previous preeclampsia	4 (4.2%)	11 (0.8%)	0.014*
Family history of DM	23 (24.0%)	154 (11.4%)	< 0.001*
Previous macrosomia	1 (1.0%)	6 (0.4%)	0.381
PCOS history	0 (0%)	7 (0.5%)	> 0.999
IVF	11 (11.5%)	146 (10.8%)	0.833
Cardiovascular disease	0 (0%)	1 (0.1%)	> 0.999
Chronic hypertension	6 (5.2%)	16 (1.2%)	0.010*

Data are presented as median (interquartile range) or number (%).

GDM, gestational diabetes mellitus; BMI, body mass index; DM, diabetes mellitus; PCOS, polycystic ovarian syndrome; IVF, in vitro fertilization.

*P < 0.05 was considered statistically significant.

### 3.2 First-trimester screening parameters of pregnant women

**Table 3** shows comparisons of first-trimester screening parameters between the GDM and non-GDM groups. There were no significant differences in crown-lump length, nuchal translucency, uterine artery PI, MAP, and free β-hCG level between the two groups. However, significantly higher MAP (86.3 vs. 81.7 mmHg, *P* < 0.001), lower PAPP-A level (5.01 vs. 5.73 IU/L, *P* < 0.001), and lower PLGF level (39.88 vs. 41.81 pg/mL, *P* = 0.044) were noted in the GDM group compared to the non-GDM group.

**Table 3:** Parameters of first-trimester screening.

	GDM (N = 96)	Non- GDM (N = 1356)	*P* value
Gestational age at scan (weeks)	12.6 (12.3–12.9)	12.7 (12.4–13.0)	0.570
CRL (mm)	66.2 (62.6–70.0)	66.5 (62.9–70.2)	0.361
NT (mm)	1.8 (1.6–2.0)	1.8 (1.6–2.0)	0.190
Uterine artery PI	1.52 (1.30–1.98)	1.57 (1.29–1.89)	0.956
MAP (mmHg)	86.3 (79.7–93.7)	81.7 (75.7–88.0)	< 0.001*
PAPP-A (IU/L)	5.01 (3.26–6.34)	5.73 (3.99–7.97)	< 0.001*
Free β-hCG (IU/L)	38.00 (25.80–61.23)	40.85 (27.90–61.40)	0.261
PLGF (pg/mL)	39.88 (27.22–50.69)	41.81 (29.43–55.60)	0.044*

Data are presented as median (interquartile range).

GDM, gestational diabetes mellitus; CRL, crown-lump length; NT, nuchal translucency; PI, pulsatility index; MAP, mean arterial pressure; PAPP-A, pregnancy-associated plasma protein A; β-hCG, beta-human chorionic gonadotropin; PLGF, placental growth factor.

*P < 0.05 was considered statistically significant.

### 3.3 ROC curve and logistic regression analyses of parameters related to GDM

**Figure 2:**
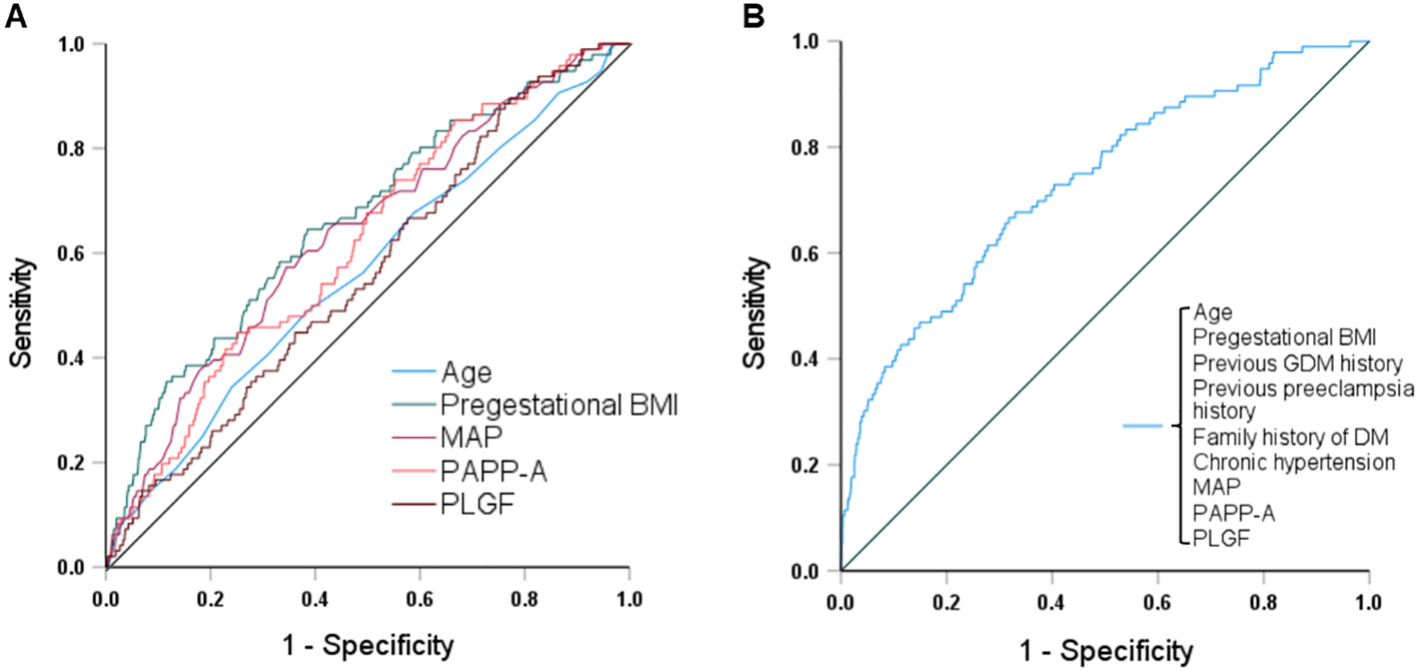
Receiver operating characteristic curves and the areas under the curves (AUCs) of **(A)** age, pregestational BMI, MAP, PAPP-A and PLGF, and **(B)** combined maternal factors and biomarkers to predict GDM. The AUCs for age, pregestational BMI, MAP, PAPP-A, PLGF, and the combination were 0.56, 0.66, 0.64, 0.62, 0.56, and 0.73, respectively. BMI, body mass index; MAP, mean arterial pressure; PAPP-A, pregnancy-associated plasma protein A; PLGF, placental growth factor; GDM, gestational diabetes mellitus; DM, diabetes mellitus.

**Figure 2** and **Table 4** show the results of ROC curve analyses of individual and combined parameters associated with GDM. The area under the curve of combined parameters to predict the development of GDM was 0.73 (95% confidence interval [CI] 0.68 - 0.79, *P* < 0.001), with a sensitivity of 0.70 and specificity of 0.63. The formula for predicting GDM was as follows:

$\begin{array}{c} P=1/[1+exp(-8.148+0.057\times \text{age}+0.011\times \\ \text{pregestational BMI}+1.752\times \text{previous GDM history}+0.95\times \\ \text{previous preeclampsia history}+0.756\times \text{family history of DM}+\\ 0.025\times \text{chronic hypertension}+0.036\times \text{MAP}-0.09\times \text{PAPP}-\\ \text{A}-0.001\times \text{PLGF})] \end{array}$

**Table 4:** ROC curve analyses of various parameters associated with GDM.

	AUC	95% CI	Cut-off value	Sensitivity	Specificity	*P* value
Age	0.56	0.50 - 0.62	32.5 years	0.56	0.51	0.040*
Pregestational BMI	0.66	0.60 - 0.72	22.45 kg/m^2^	0.65	0.61	< 0.001*
MAP	0.64	0.58 - 0.69	84.17 mmHg	0.60	0.62	< 0.001*
PAPP-A	0.62	0.56 - 0.67	4.95 IU/L	0.61	0.50	< 0.001*
PLGF	0.56	0.51 - 0.62	40.64 pg/mL	0.53	0.52	0.044*
Combination^a^	0.73	0.68 - 0.79		0.70	0.63	< 0.001*

ROC, receiver operating characteristic; GDM, gestational diabetes mellitus; BMI, body mass index; MAP, mean arterial pressure; PAPP-A, pregnancy-associated plasma protein A; PLGF, placental growth factor; AUC, area under the curve; CI, confidence interval.

*P < 0.05 was considered statistically significant.

a Including age, pregestational BMI, MAP, PAPP-A, PLGF, previous gestational diabetes mellitus and preeclampsia history, family history of diabetes mellitus, and chronic hypertension (as shown in **Figure 2B**).

Multivariate logistic regression analyses still revealed significant differences in pregestational BMI (adjusted odds ratio [aOR] 1.03, 95% CI 1.01 - 1.06; *P* = 0.012), previous GDM (aOR 9.97, 95% CI 3.92 - 25.37; *P* < 0.001), family history of DM (aOR 2.36, 95% CI 1.39 - 4.02; *P* = 0.001), MAP (aOR 1.17, 95% CI 1.07 - 1.27; *P* < 0.001), and PAPP-A level (aOR 0.91, 95% CI 0.83 - 1.00; *P* = 0.040) (**Table 5**). The Hosmer-Lemeshow test demonstrated that the model exhibited an excellent discrimination ability (chi-square = 3.089, df = 8, *P* = 0.929).

**Table 5:** Univariable and multivariable logistic regression analyses of individual parameters associated with GDM.

	Univariable logistic regression			Multivariable logistic regression		
	Unadjusted OR	95% CI	*P* value	Adjusted OR	95% CI	*P* value
Age	1.05	1.01 - 1.10	0.024*	1.05	1.00 - 1.10	0.073
Pregestational BMI	1.11	1.07 - 1.15	< 0.001*	1.03	1.01 - 1.06	0.012*
Previous GDM history	15.82	6.67 - 37.54	< 0.001*	9.97	3.92 - 25.37	< 0.001*
Previous preeclampsia history	5.32	1.66 - 17.02	0.005*	2.79	0.66 - 11.83	0.163
Family history of DM	2.46	1.50 - 4.05	< 0.001*	2.36	1.39 - 4.02	0.001*
Chronic hypertension	4.60	1.65 - 12.84	0.004*	0.78	0.19 - 3.21	0.734
MAP	1.05	1.03 -1.07	< 0.001*	1.17	1.07 - 1.27	< 0.001*
PAPP-A	0.85	0.79 - 0.92	< 0.001*	0.91	0.83 - 1.00	0.040*
PLGF	0.99	0.97 - 1.00	0.012*	0.99	0.98 - 1.01	0.262

GDM, gestational diabetes mellitus; BMI, body mass index; DM, diabetes mellitus; MAP, mean arterial pressure; PAPP-A, pregnancy-associated plasma protein A; PLGF, placental growth factor; OR, odds ratio; CI, confidence interval.

*P < 0.05 was considered statistically significant.

## 4 Discussion

First-trimester aneuploidy and preeclampsia screening are widely used in pregnant women. However, GDM screening is not commonly performed in the first trimester of pregnancy. Since Chinese women are a high-risk ethnic group for GDM, the aim of this study was to examine the associations between first-trimester prenatal screening biomarkers and maternal characteristics in relation to GDM in Chinese women. We found that combining maternal factors (age, pregestational BMI, history of GDM, history of preeclampsia, family history of DM, chronic hypertension, and MAP) and prenatal screening biomarkers (PAPP-A and PLGF) could identify 73% of cases of GDM. Moreover, higher maternal pregestational BMI, history of GDM, family history of DM, higher maternal MAP, and lower PAPP-A level were independently associated with developing GDM.

Previous studies on the association between first-trimester prenatal screening biomarkers (PAPP-A, free β-hCG and PLGF) and GDM have shown inconsistent results. Most of these studies demonstrated an association between a lower first-trimester PAPP-A level with the development of GDM (8, 10, 13–19, 21–23, 26, 27), however only two studies reported an association between a lower first-trimester free β-hCG level and the development of GDM (8, 13). A meta-analysis conducted in 2018 found that women diagnosed with GDM had lower levels of both PAPP-A and free β-hCG in the first trimester (32). Furthermore, some studies have reported that an increased first-trimester PLGF level could predict the development of GDM (17, 18), whereas others have not found this association (25, 26). Conversely, several animal experiments demonstrated an association between a lower PLGF level and the development of GDM (33–35). In the present study, lower levels of first-trimester PAPP-A and PLGF, but not free β-hCG, were associated with developing GDM, and our results are consistent with some of the previous studies (10, 14–16, 19, 21–23, 27) and animal experiments (33–35).

First-trimester biomarkers are influenced by ethnicity, which may also have contributed to the discrepant results in the previous studies. Asian women have been reported to have 17% and 4% higher levels of PAPP-A and free β-hCG, respectively (28), but an 11% lower level of PLGF than Caucasian women (29). Few studies have investigated the associations between PAPP-A and free β-hCG levels with the development of GDM in Chinese women (20, 23), and none have discussed the correlation between PLGF level and GDM in a Chinese population. Cheuk et al. prospectively studied 520 Chinese women, of whom 169 (32.5%) were diagnosed with GDM using the World Health Organization 1999 criteria, and they concluded that first-trimester PAPP-A and free β-hCG could not predict GDM (20). However, Xiao et al. performed a case control study of 599 women with GDM and 986 without GDM using the IADPSG criteria, and concluded that PAPP-A, but not free β-hCG, was independently associated with the development of GDM (23), which agrees with our study. Moreover, our study is the first to demonstrate an association between a lower level of PLGF with the development of GDM in Chinese women.

PAPP-A, an insulin growth factor (IGF)-dependent insulin growth factor binding protein (IGFBP) protease, can modulate IGF bioavailability through the proteolysis of IGFBP-2, 4, and 5. The availability of IGF is influenced by maternal adipose tissue physiology and systemic glucose regulation (36). Decreased PAPP-A expression is linked to reduced IGF availability due to restricted IGFBP proteolysis, a process typically augmented during pregnancy to meet the elevated demand for bioactive IGF (37). PLGF, a member of the vascular endothelial growth factor family, can promote islet endothelial cells to release growth factors to trigger beta-cell growth, and failure of this response may lead to impaired glucose tolerance and the development of GDM (33–35). In addition, PLGF can activate brown adipose tissue, which improves glucose homeostasis and mitigates insulin resistance, and downregulation of PLGF may contribute to the reduced brown adipose tissue activity in GDM (38). From these points of view, first-trimester PAPP-A and PLGF could be early biomarkers for predicting the development of GDM. In this study, we found that downregulation of first-trimester PAPP-A and PLGF was associated with the development of GDM, and that a lower level of first-trimester PAPP-A was independently associated with the development of GDM after logistic regression analysis. Some discrepancies with earlier reports may be attributed to differences in ethnic populations and the diagnostic criteria for GDM. Further comparative analyses and jointly conducted studies are necessary to better understand and clarify these differences.

Previous studies have validated various risk factors for GDM, including older age, higher BMI, history of GDM, and family history of DM; however, using historic risk factors in screening would fail to identify approximately one half of women with GDM (1). In comparison, a screening strategy combining maternal risk factors and related biomarkers has been shown to increase the predictive ability for GDM (14, 17). We also found that chronic hypertension was related to the development of GDM, and that elevated MAP was independently associated with the development of GDM, which is consistent with previous studies (39, 40). Lao et al. reported a positive correlation between the incidence of GDM and first-trimester systolic BP in high-risk Chinese women (39). In addition, Hedderson et al. demonstrated that women with hypertension before or during the first trimester of pregnancy had a twofold increased risk of developing GDM (40). Hypertension is associated with insulin resistance by altering glucose metabolism in peripheral tissues (41), which is one of the pathogenetic mechanisms of GDM.

Our study has several strengths. First, we specifically examined the relationships between three first-trimester biomarkers (PAPP-A, free β-hCG, and PLGF) and GDM in a cohort of Chinese women. To the best of our knowledge, this is the first study to investigate the associations between these three distinct prenatal screening biomarkers and GDM within a Chinese population. Second, the predictive method for GDM is cost-effective as it is based on maternal characteristics and biomarkers which are currently used in prenatal screening. There are also several limitations to this study. First, we lacked data of first-trimester blood glucose and hemoglobin A1c, which are not routinely examined in our hospital. Second, we excluded women with current preeclampsia to minimize the impact of preeclampsia-related downregulation of PAPP-A and PLGF. Consequently, the association between GDM and preeclampsia could not be investigated in this study. Third, GDM was diagnosed using either the one-step or two-step criteria depending on the attending physician’s discretion, potentially introducing bias into this study.

## 5 Conclusions

Our results revealed that lower levels of PAPP-A and PLGF were significantly associated with the development of GDM, with PAPP-A proving to be a more effective predictor than PLGF for the early detection of GDM. Importantly, our findings suggest that the integration of first-trimester biomarkers with maternal characteristics could serve as a valuable tool for predicting the risk of GDM. These findings provide essential insights into the early identification and management of GDM in Chinese women, offering opportunities for timely interventions and personalized care. Healthcare providers should advise pregnant women at high risk of developing GDM on early lifestyle interventions, such as dietary control and exercise, to reduce the likelihood of developing GDM.

## Data availability statement

The original contributions presented in the study are included in the article/supplementary material. Further inquiries can be directed to the corresponding author.

## Ethics statement

The studies involving humans were approved by the Institutional Review Board of MacKay Memorial Hospital (IRB no. 22MMHIS318e). The studies were conducted in accordance with the local legislation and institutional requirements. The ethics committee/institutional review board waived the requirement of written informed consent for participation from the participants or the participants’ legal guardians/next of kin because the retrospective nature of this study based on patient charts. All personal identifiers were anonymized prior to analysis.

## Author contributions

Y-TL: Conceptualization, Formal analysis, Writing – original draft. C-PC: Formal analysis, Methodology, Writing – review & editing. F-JS: Formal analysis, software, Writing – review & editing. Y-YC: Data curation, Writing – review & editing. L-KW: Data curation, Writing – review & editing. C-YC: Conceptualization, Methodology, Writing – review & editing.
